# *Aedes albopictus* salivary adenosine deaminase is an immunomodulatory factor facilitating dengue virus replication

**DOI:** 10.1038/s41598-023-43751-1

**Published:** 2023-10-04

**Authors:** Xiaohui Mu, Zimin Lin, Yu Sun, Lu Chen, Qingqiao Lv, Cejuan Ji, Xiaoyuan Kuang, Weiyi Li, Zhengling Shang, Jinzhi Cheng, Ying Nie, Zhiqiang Li, Jiahong Wu

**Affiliations:** 1 Department of Parasitology, Provincial Key Laboratory of Modern Pathogen Biology, College of Basic Medical Sciences, Guizhou Medical University, Guiyang, 550025 Guizhou China; 2 Department of Reproductive Medicine, People’s Hospital of Anshun City Guizhou Province, Anshun, 561000 Guizhou China; 3 The Affiliated Hospital of Guizhou Medical University, Guiyang, 550004 Guizhou China; 4 Xi’an Peihua University, Xi’an, 710065 Shaanxi China; 5 Department of Medical Technology, Guiyang Healthcare Vocational University, Guiyang, Guizhou China; 6 Department of Immunology, College of Basic Medicine, Guizhou Medical University, Guiyang, 550025 China

**Keywords:** Cell biology, Immunology, Microbiology, Molecular biology, Diseases, Pathogenesis

## Abstract

The Asian tiger mosquito, *Aedes albopictus*, is an important vector for the transmission of arboviruses such as dengue virus (DENV). Adenosine deaminase (ADA) is a well-characterized metabolic enzyme involved in facilitating blood feeding and (or) arbovirus transmission in some hematophagous insect species. We previously reported the immunologic function of ADA by investigating its effect on mast cell activation and the interaction with mast cell tryptase and chymase. The 2-D gel electrophoresis and mass spectrometry analysis in the current study revealed that ADA is present and upregulated following mosquito blood feeding, as confirmed by qRT-PCR and western blot. In addition, the recombinant ADA efficiently converted adenosine to inosine. Challenging the Raw264.7 and THP-1 cells with recombinant ADA resulted in the upregulation of IL-1β, IL-6, TNF-α, CCL2, IFN-β, and ISG15. The current study further identified recombinant ADA as a positive regulator in NF-κB signaling targeting TAK1. It was also found that recombinant *Ae. albopictus* ADA facilitates the replication of DENV-2. Compared with cells infected by DENV-2 alone, the co-incubation of recombinant ADA with DENV-2 substantially increased IL-1β, IL-6, TNF-α, and CCL2 gene transcripts in Raw264.7 and THP-1 cells. However, the expression of IFN-β and ISG15 were markedly downregulated in Raw264.7 cells but upregulated in THP-1 cells. These findings suggest that the immunomodulatory protein, *Ae. albopictus* ADA is involved in mosquito blood feeding and may modulate DENV transmission via macrophage or monocyte-driven immune response.

## Introduction

Arthropod mosquito is a significant human pathogen vector as diseases caused by mosquito-borne viruses have a significant negative impact on global health. Dengue virus (DENV) is the most prevalent mosquito-borne virus, affecting approximately half of the global population living in dengue-endemic countries. DENV infects 390 million people a year worldwide^1^. *Aedes aegypti* is known as the primary vector for DENV transmission. The role of *Ae. albopictus* in causing DENV outbreaks has grown bigger due to rapid changes in their distribution, posing a profound threat to public health and burdening the global economy^2–4^. DENV is transmitted into vertebrates via the saliva of infected mosquitoes during blood feeding. Numerous studies have reported that mosquito saliva contains a complex and diverse mixture of anti-hemostatic, anti-inflammatory, and immunomodulatory compounds that facilitate blood meal acquisition and arbovirus transmission^5,6^.

Mosquito saliva contains proteins that are immunogenic to humans, sometimes eliciting severe allergic responses^7,8^. By injecting a mixture of pharmacologically active proteins into the host during blood-feeding, mosquitos can cause type I hypersensitivity and induce strong immune responses involving macrophages and monocytes^9,10^. Immunogenic roles of salivary proteins derived from *Ae. albopictus* was first screened in 2013^11^. However, current studies have only partially elucidated the functions of these proteins. AlboD7L1 reportedly exerts anti-hemostatic and anti-inflammatory effects by inhibiting platelet aggregation and neutrophil recruitment in the host during mosquito blood feeding^12^. Meanwhile, 34k2 was found to have the ability to induce the production of IgG antibodies, a promising candidate marker for detecting human exposure to *Ae. albopictus*^13,14^. This protein may also be involved in mast cell-driven immune responses following mosquito biting, as demonstrated by its interaction with mast cells, leading to the release of specific proteases tryptase, and chymase^15^.

When it comes to the effect of mosquito saliva on DENV, many researchers have proved the role of proteins in the saliva in DENV replication. In *Ae. aegypti* models infected with DENV, the virus titer, and systemic infection were magnified by mosquito saliva^16–18^. Several salivary components have been identified as the factors affecting the infection or transmission of arboviruses. A saliva protein of *Ae. aegypti* called AsSG34 reportedly enhanced the transmission of DENV to the mammalian host^19^. AaVA-1, a saliva protein of *Ae. aegypti* reportedly induced the enhancement of DENV replication in macrophages^20^. On the contrary, a saliva protein of *Ae. aegypti* called D7 was reported to inhibit DENV infection^21^. Although studies have associated *Ae. albopictus* with the transmission of DENV^22–24^, very few investigated the effect of salivary proteins on DENV infection in *Ae. albopictus* model.

The genome sequences of several model organisms and humans reveal the presence of more than one adenosine deaminase (ADA) gene in metazoan^25^. ADA catalyzes the deamination of adenosine and deoxyadenosine into their respective inosine nucleosides. ADA is an important immune regulatory molecule in humans, which plays an important role in the maturation and maintenance of the immune system. ADA deficiency leads to an accumulation of toxic purine degradation by-products, primarily affecting lymphocytes, leading to severe combined immunodeficiency (SCID) caused by adenosine deaminase deficiency^26,27^. Higher *Diptera* carry an unusually high number of ADA genes compared with other organisms^25,28^. ADA genes of mosquitos were first found in *Culex quinquefascia*-*tus* and *Ae. aegypti*, whereby their activity was measured in the latter^28^*.* High expression of ADA was reported in the salivary glands of *Ae. aegypti*, which may be vital for blood feeding and degrading purinergic mediators of hemostasis and inflammation^29^. Additionally, evidence has demonstrated the role of ADA in virus replication in *Ae. aegypti*^30,31^. However, the underlying mechanisms of ADA are not fully characterized. A significant expression level of ADA gene in salivary glands of female *Ae. albopictus* was reported*,* which is currently regarded as a putative secreted salivary protein according to transcriptomic and proteomic analysis^32^. However, no studies have investigated the activity and function of ADA in *Ae. albopictus.*

In this work, we screened and identified the upregulated salivary protein, ADA, from the salivary glands of blood meal in female *Ae. albopictus* model. Studies have revealed the enzymatic activity of ADA of *Ae. albopictus*, particularly its role as an immunomodulatory factor involved in the inflammatory response. This response was induced by mosquito blood feeding, which triggers monocyte or macrophage-driven immune response, demonstrating a vital role in DENV transmission through modulating the release of associated cytokines.

## Results

### *Ae. albopictus* ADA is involved in blood-feeding

**Figure 1:**
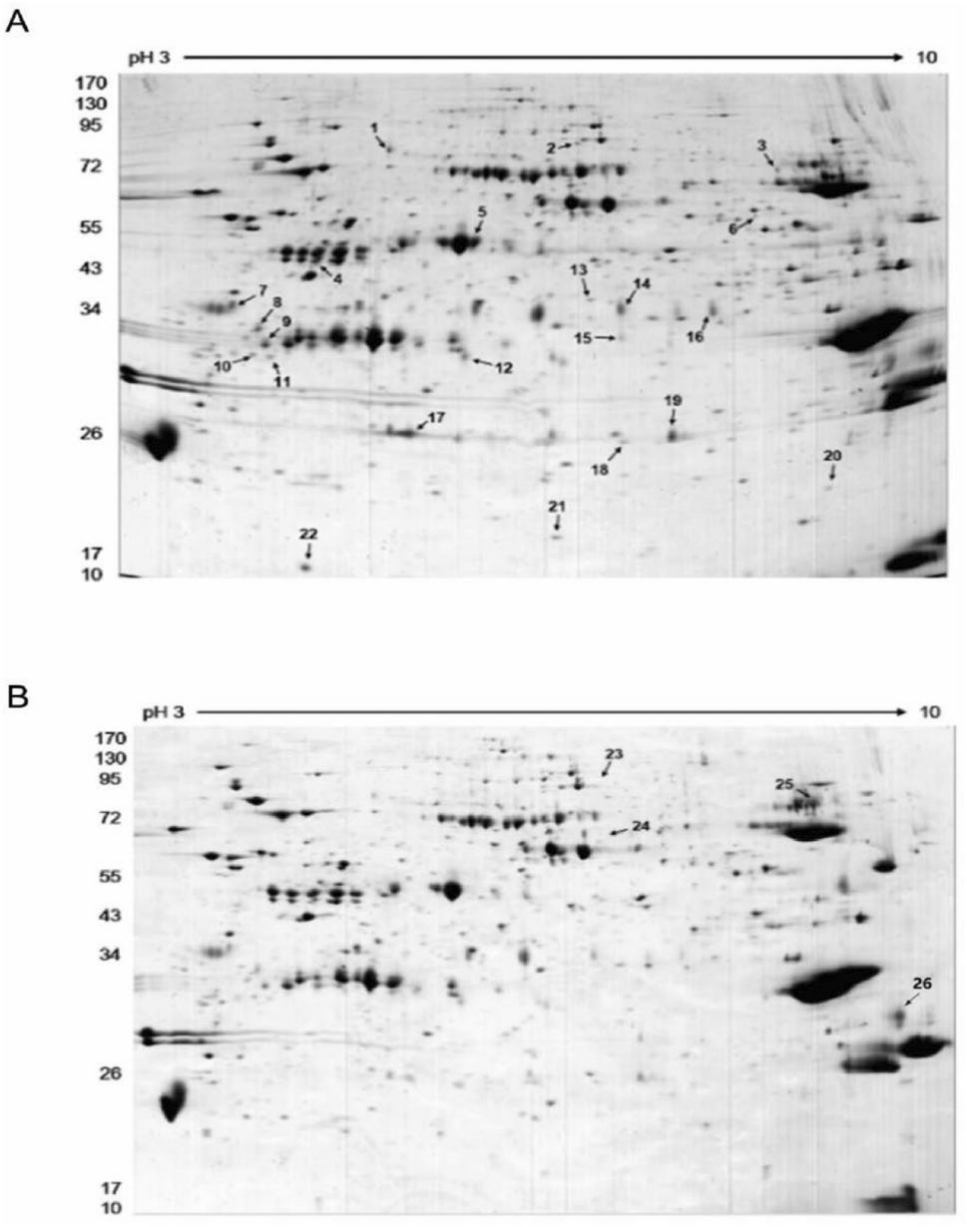
Two-dimensional gel analysis of differentially expressed salivary gland proteins in female *Ae. albopictus* mosquitoes with or without a blood meal. Salivary glands of 300 μg extracted from five-day-old unfed or blood-fed mosquitoes were loaded onto 3–10 NL immobilins (24 cm) before SDS-PAGE separation on a 12.5% precast gel. Protein spots were stained with Coomassie Blue R-250 for 2 h. Molecular weight markers are indicated on the left in kDa. Isoelectric points (pI) are indicated at the top. Gel profiles were compared using Image Master Platinum software. The spots corresponding to the differentially expressed proteins are indicated by arrows (fold change of > 2.0; Anova of < 0.05). (**A**) 2D-gel separation of salivary proteins extracted from unfed female mosquitoes. (**B**) 2D-gel separation of salivary proteins extracted from blood-fed female mosquitoes. Data are representative of three independent experiments.

*Aedes albopictus* triggers the host immune system by injecting salivary proteins. To find the key target proteins related to blood feeding, a comparative analysis of salivary protein profiles between female models with and without blood meal was performed using 2-D gel electrophoresis (2-DE) (Fig. 1). Analysis of the gels using 2D Image Platinum software revealed 547 spots in the unfed female salivary gland, mainly distributed between 20 and 130 kDa. Comparisons between the unfed and blood-fed groups revealed 26 protein spots with a saliency of more than two, which were further characterized. Out of these 26 spots, 22 spots were downregulated (Fig. 1A) and 4 spots were upregulated (Fig. 1B) in the blood-fed group compared with the unfed group. The results suggest that these salivary proteins may be involved in mosquito blood-feeding.

**Figure 2:**
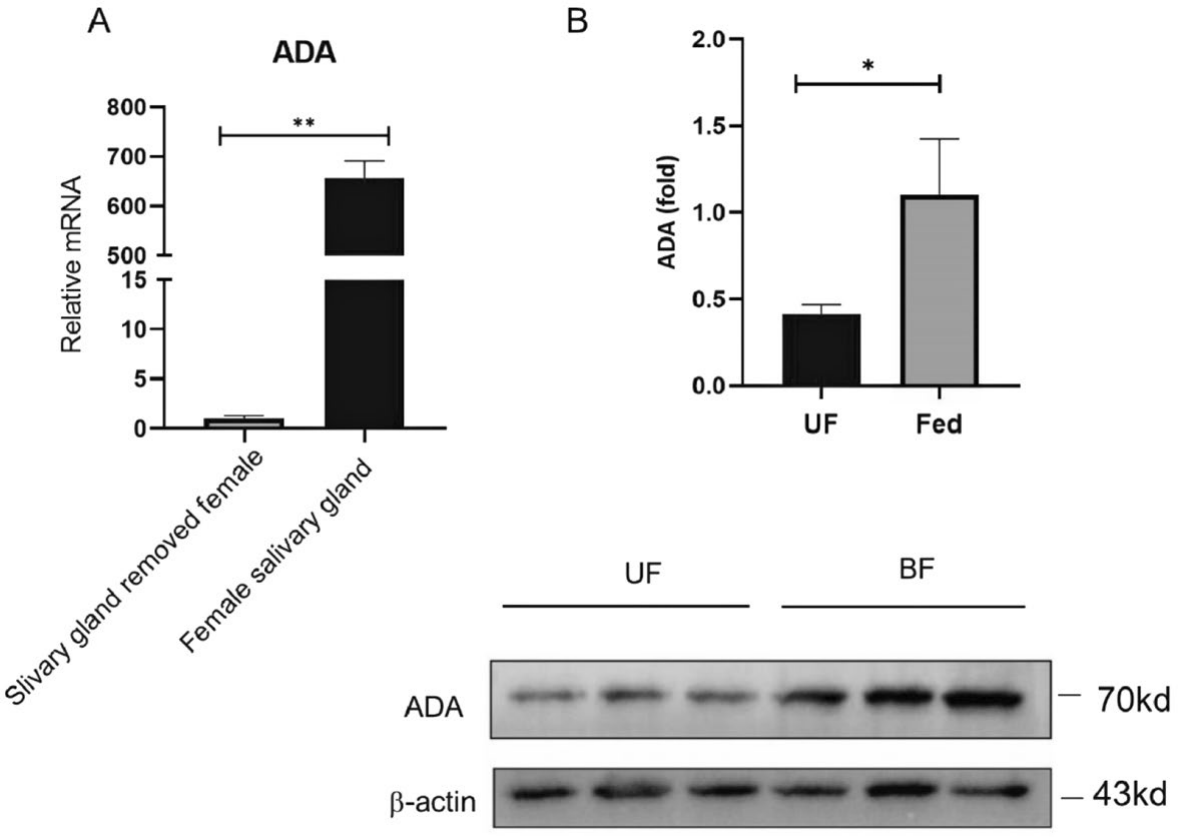
ADA is predominantly present in salivary glands and is upregulated following a mosquito blood meal. (**A**) Total mRNAs were separately extracted from salivary glands or female mosquitoes with salivary glands removed; the mRNA level of ADA was analyzed by qRT-PCR. (**B**) Salivary glands were collected from five-day-old female mosquitoes with (BF) or without (UF) blood-fed and the expression of salivary ADA was detected by immunoblotting with anti-ADA antibody (down). The signal intensity of each protein corresponds to the β-actin (up). n = 3. *p < 0.05; **p < 0.01.

To identify the key salivary gland proteins, 26 variant spots were excised and digested by trypsin and analyzed by MALDI-TOF/TOF mass spectrometry. As shown in Table 1, there were 23 major protein spots, of which ADA exhibited the largest fold expression among these upregulated proteins. However, the other three spots were not detected, possibly due to the high complexity of the spots in the sample. To validate the upregulated ADA in salivary glands of the blood-fed group, its expression in salivary glands of female *Ae. albopictus* was first confirmed (Fig. 2A) via immunoblotting using an anti-ADA antibody. The results demonstrate the upregulation of ADA in the salivary glands of the blood-fed group following the feeding (Fig. 2B). These results imply the role of ADA in mosquito blood-feeding.

**Table 1:** Salivary gland proteins of unfed and blood-fed female mosquitoes identified by MALDI-TOF MS analysis.

Spot number	Protein name	Accession number	PI	Molecular	Match peptide
24	Putative adenosine deaminase	gi|56417438	6.27	61,652.6	27
23	Glutamate semialdehyde dehydrogenase	gi|157114403	6.76	87,531.2	30
25	Salivary apyrase	gi|303324515	8.17	63,167.8	22
18	Mannosyltransferase	gi|157131338	6.29	24,266.9	5
3	Putative 56 kDa salivary secreted protein	gi|56417492	8.4	58,740	9
9	Angiopoietin-like salivary protein	gi|56417466	5.06	32,411.6	8
12	Angiopoietin-like protein variant	gi|94468352	5.74	33,515.4	8
10	angiopoietin-like salivary protein	gi|56417466	5.06	32,411.6	9
1	Similar to *Aedes aegypti* 34 kDa salivary secreted protein 34k-2	gi|56417498	5.95	36,848.9	19
8	Angiopoietin-like salivary protein	gi|56417466	5.06	32,411.6	12
14	Putative secreted protein	gi|18568298	5.12	33,983.3	4
19	Angiopoietin-like protein 2	gi|56417464	6.34	27,227	12
7	Salivary purine nucleosidase	gi|56417432	4.88	39,588.9	11
11	Angiopoietin-like salivary protein	gi|56417466	5.06	32,411.6	7
17	Salivary secreted antigen-5 precursor AG5-3	gi|56417472	9.05	30,030.4	2
4	Putative salivary serpin	gi|56417454	5.54	47,961.4	14
21	Transcription factor BTF3a	gi|56417472	6.32	17,102	4
22	Initiation factor 5a	gi|108880628	5.3	17,854.7	9
2	Protein transport protein sec23	gi|157116181	6.21	87,942.3	19
6	UDP-glucose pyrophosphorylase	gi|61608458	6.69	58,219.2	20
5	salivary serpin putative anticoagulant	gi|56417462	6.15	47,024.5	18
13	Alcohol dehydrogenase [*Aedes aegypti*]	gi|108872591	6.31	38,871.8	11
20	Calponin/transgelin	gi|108877213	8.29	20,936.4	15

### *Aedes albopictus* ADA shows enzymic activity

**Figure 3:**
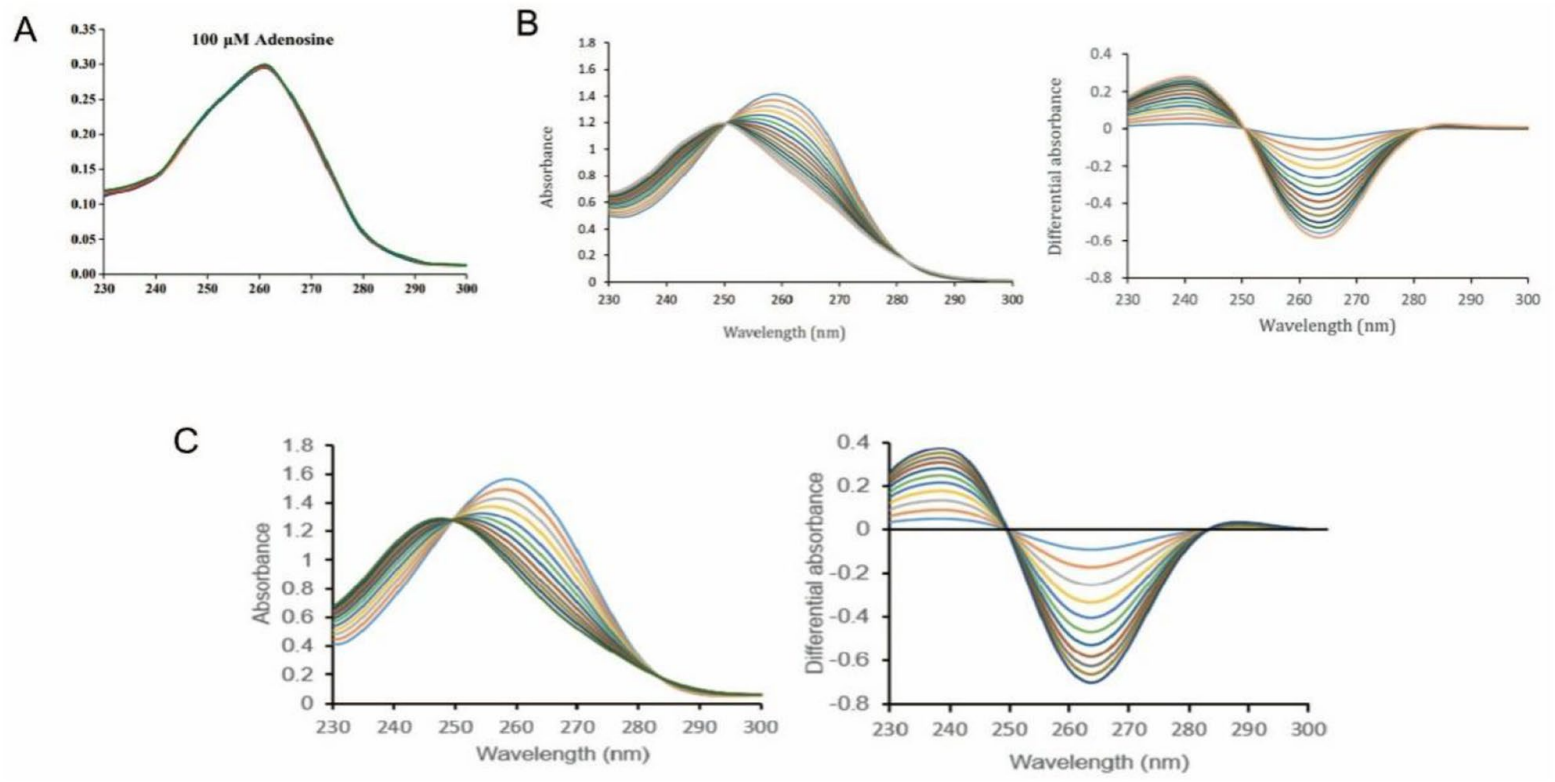
Enzymatic activity of *Ae. albopictus* ADA. ADA activity was measured by the spectrophotometric method. ADA activity in salivary homogenates of adult female *Ae. albopictus*: a cuvette containing 1 mM adenosine in 100 µL of HS buffer was continuously scanned 30 times at the wavelengths ranging from 230 to 300 nm for samples without (**A**) or with (**B**) the addition of 10 μL salivary gland homogenates extracted from 20 pairs of female salivary glands at 5-day-old PE (left panel). The differential spectra (right panel) of the data in the left panel were obtained by subtracting each read from the read at time zero. (**C**) Recombinant ADA exhibiting adenosine deaminase activity: a cuvette containing 1 mM adenosine in 100 µL of HS buffer was continuously scanned 30 times at the wavelengths ranging from 230 to 300 nm following the addition of 0.67 µM recombinant ADA (left panel). The differential spectra (right panel) of the data in the left panel were obtained by subtracting each read from the read at time zero. Data are representative of three independent experiments. Colors represent different times.

To further examine the functions of ADA, ADA was expressed in prokaryotes and purified as a soluble protein in the supernatant of bacterial lysate. ADA catalyzes the deamination of adenosine to inosine and deoxyadenosine to deoxyinosine. To test the activity of ADA in the saliva of *Ae. albopictus*, soluble proteins from salivary gland homogenates of *Ae. albopictus* and the recombinant ADA （rAb-ADA）were incubated in the presence of adenosine. The changes were measured spectrophotometrically at the wavelengths from 220 to 300 nm. The substrate adenosine absorbs light at 265 nm, and the product of ADA activity, inosine, absorbs at 241 nm. Compared to the blank control (Fig. 3A), the salivary homogenate equivalent to 0.2 pairs salivary gland converted adenosine to inosine (Fig. 3B). Similarly, rAb-ADA of *Ae. albopictus* converted adenosine to inosine (Fig. 3C). The differential spectrum (Fig. 3B right panel and Fig. 3C right panel) shows the decrease of adenosine (265 nm) and the gradual increase of inosine (241 nm) in the presence of salivary gland homogenates or rAb-ADA of *Ae. albopictus*. These results demonstrate the adenosine deaminase activity in *Ae. albopictus*, proving the enzymatic activity of rAb-ADA.

### rAb-ADA induces cytokines release from monocytes and macrophages

**Figure 4:**
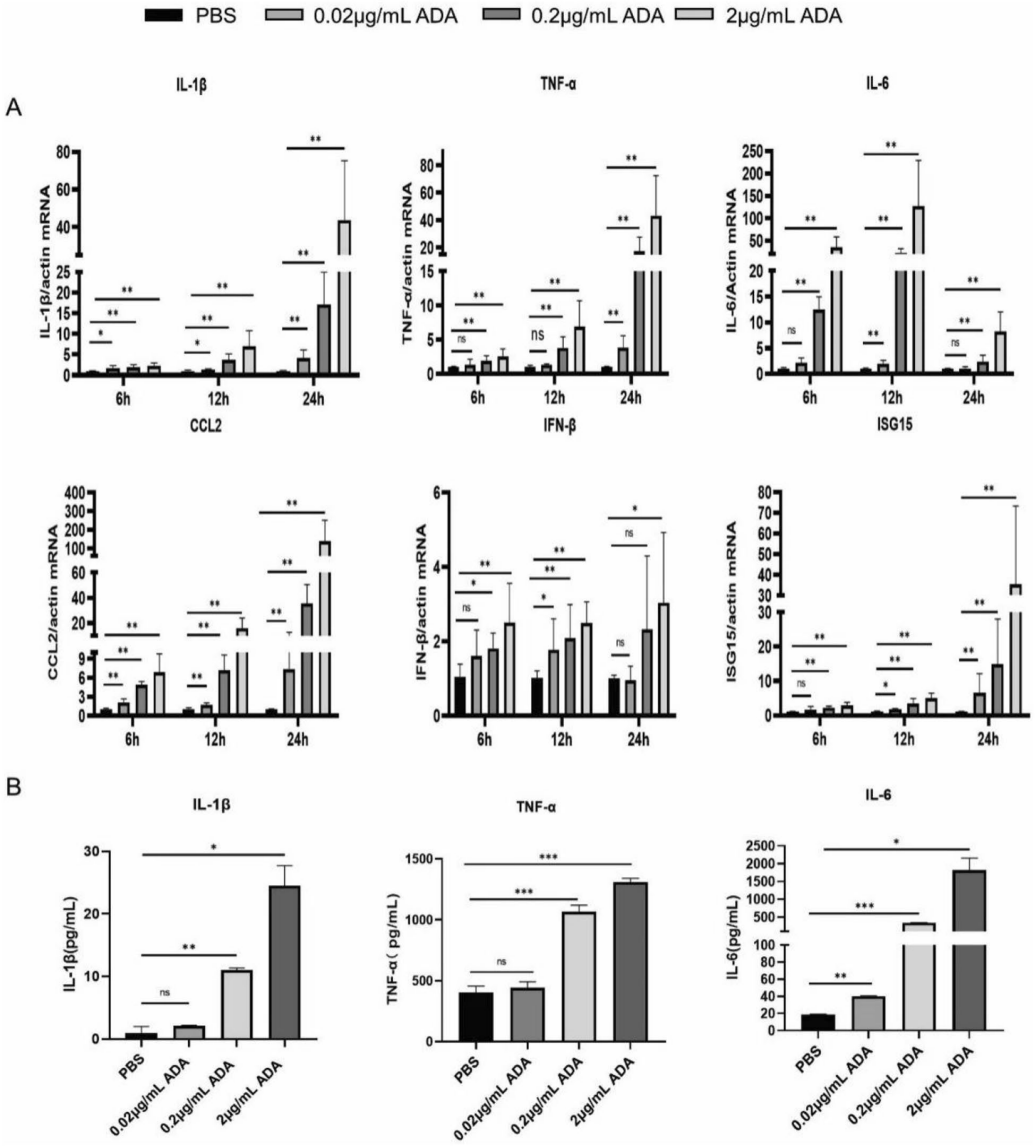
Recombinant *Ae. albopictus* ADA promotes cytokines production in vitro. (**A**) Human THP-1 cells were treated with various doses (0.02, 0.2, and 2 μg/mL) of recombinant *Ae. albopictus* ADA. RNA was isolated from the cells at various time points (6 h,12 h, and 24 h) following the stimulation, cDNA was generated, and qRT-PCR was performed to measure the mRNA level of IL-1β, IL-6, TNF-α, CCL2, IFN-β, and ISG15. The data were normalized to human actin by applying the ΔΔCT method and are presented as percentages of the average ΔΔCT value of untreated (PBS) cells. (**B**) ELISA of IL-1β, IL-6, and TNF-α in supernatants of Raw 264.7 cells treated with various doses (0.02, 0.2, and 2 μg/mL) of recombinant *Ae. albopictus* ADA at 24 h. A nonparametric Mann–Whitney test was applied for the statistical analysis. Labels: ns indicates insignificant (p > 0.05); *p < 0.05; **p < 0.01; ***p < 0.001. The data are representative of three independent experiments.

We previously demonstrated the activation of mast cells by ADA and its interaction with specific proteases in mast cells^15^. Macrophages reside in the skin dermis, whereas monocytes circulate in the blood, enabling them to directly respond to mosquito saliva proteins. Therefore, we evaluated the immunomodulatory roles of rAb-ADA in Raw264.7 and THP-1 cells by evaluating the levels of related cytokines. As depicted in the results, the expression of genes encoding cytokines, IL-1β, IL-6, TNF-α, CCL2, IFN-β, and ISG15, were significantly upregulated when challenged with rAb-ADA (Figs. 4A, S1). Similarly, the production of IL-1β, IL-6, and TNF-α in supernatants was markedly induced by rAb-ADA (Fig. 4B). These results suggest that rAb-ADA may modulate inflammatory responses following mosquito biting via the activation of macrophage or monocyte.

### rAb-ADA activates the NF-κB signaling pathway by targeting TAK1

**Figure 5:**
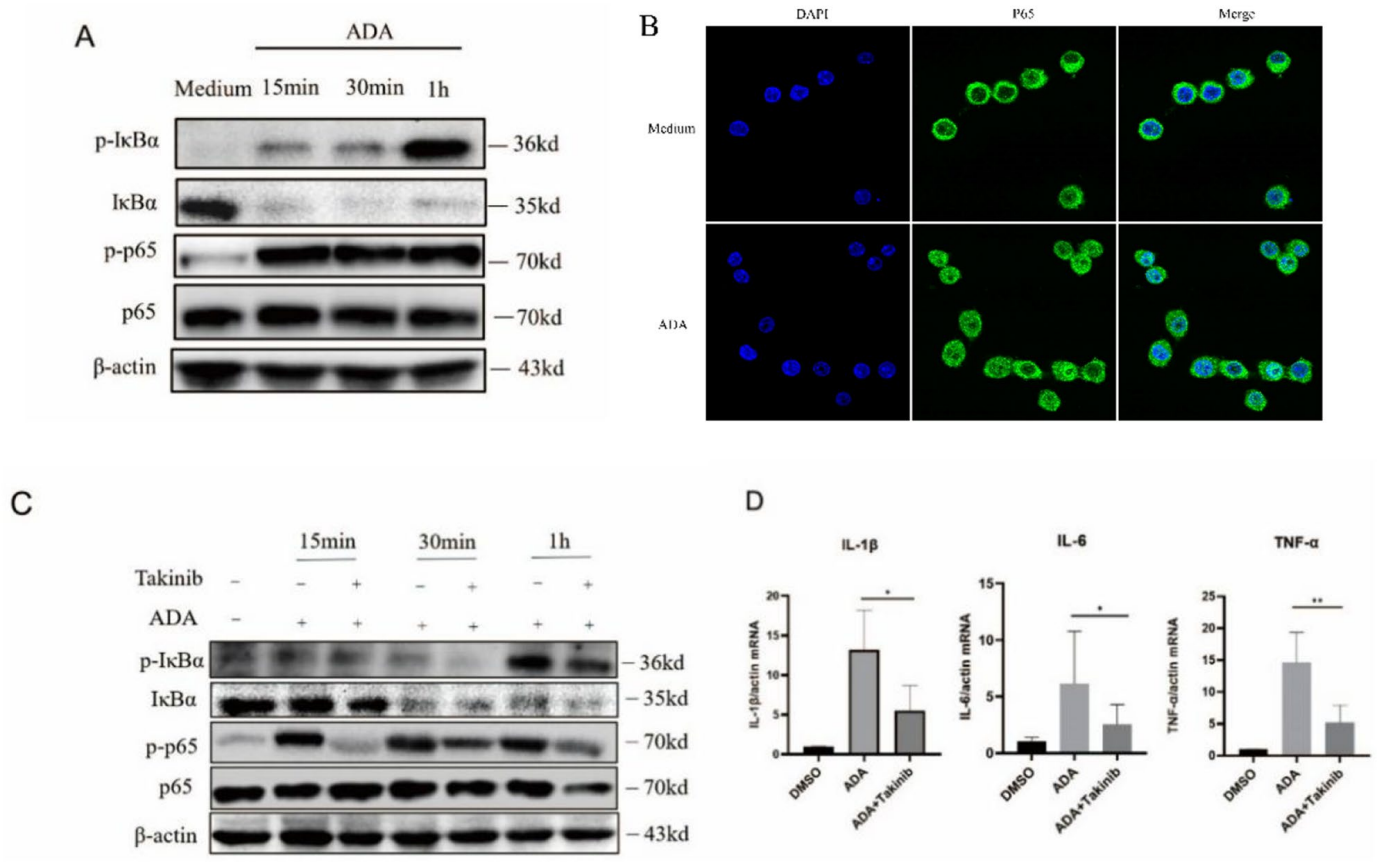
Recombinant *Ae. albopictus* ADA activates the NF-κB pathway by targeting Takinib. (**A**) Immunoblot analysis of phosphorylated IκBα(p-IκBα), total proteins of IκBα(IκBα), phosphorylated p65(p-p65), and p65 in human THP-1 cells from untreated (Medium) or treated with ADA (2 μg /mL) groups at various time points (15 min, 30 min, and 1 h). (**B**) Confocal microscopy of p65 in mouse Raw264.7 cells from untreated (Medium) or treated with ADA (2 μg /mL) groups at 1 h. (**C**) Human THP-1 cells treated with the inhibitors, Takinib (10 μM) or DMSO, for 3 h before being stimulated with ADA (2 μg/mL) at various time points (15 min, 30 min, and 1 h); p-IκBα, IκBα, p-p65and p65 were analyzed via Immunoblot assay. (**D**) Human THP-1 cells were treated with the inhibitors, Takinib (10 μM) or DMSO, for 3 h before stimulation with ADA (2 μg/mL) for 24 h; RNA was then isolated from the cells, cDNA was generated, and qRT-PCR was performed to measure the mRNA level of IL-1β, IL-6, and TNF-α. A nonparametric Mann–Whitney test was applied for the statistical analysis. *p < 0.05; **p < 0.01. The data are representative of two independent experiments.

The nuclear factor, NF-κB, is a prototypical proinflammatory signaling pathway that induces the expression of proinflammatory genes including cytokines, chemokines, etc.^33^. Therefore, we investigated the regulatory effects of rAb-ADA on the NF-κB pathway by detecting the phosphorylation levels of the p65 and IκBα, as well as the nuclear translocation of p65. Immunoblot assay revealed that rAb-ADA induced the degradation of IκBα and upregulated the phosphorylation of the p65 and IκBα (Fig. 5A). Notably, immunofluorescence analysis confirmed the role of rAb-ADA in promoting the localization of p65 in the nucleus of Raw264.7(Fig. 5B).

Furthermore, we analyzed the effects of NF-κB inhibitor, BAY11–7082, and TAK1 inhibitor, Takinib, on rAb-ADA-mediated NF-κB activation. BAY11–7082, a nuclear factor-κB inhibitor irreversibly inhibits the phosphorylation and degradation of IκBα^34^. Takinib, a selective TAK1 inhibitor, inhibits TAK1 activation^35^. TAK1 phosphorylates IKKβ through its proximity to the IKK complex, which leads to NF-κB activation via the phosphorylation and subsequent degradation of IκB proteins^36^. The results demonstrate a significant downregulation of p-IκBα and p-p65 by Takinib (Fig. 5C) and BAY11–7082 (Fig. S2A) treatment at 1 h. The level of mRNA corresponding to IL-1β, IL-6, and TNF-α was significantly suppressed by Takinib (Fig. 5D), but not by BAY11–7082 (Fig. S2B). In general, the results demonstrate that rAb-ADA activates NF-κB signaling pathway by targeting TAK1.

### rAb-ADA promotes DENV-2 replication

**Figure 6:**
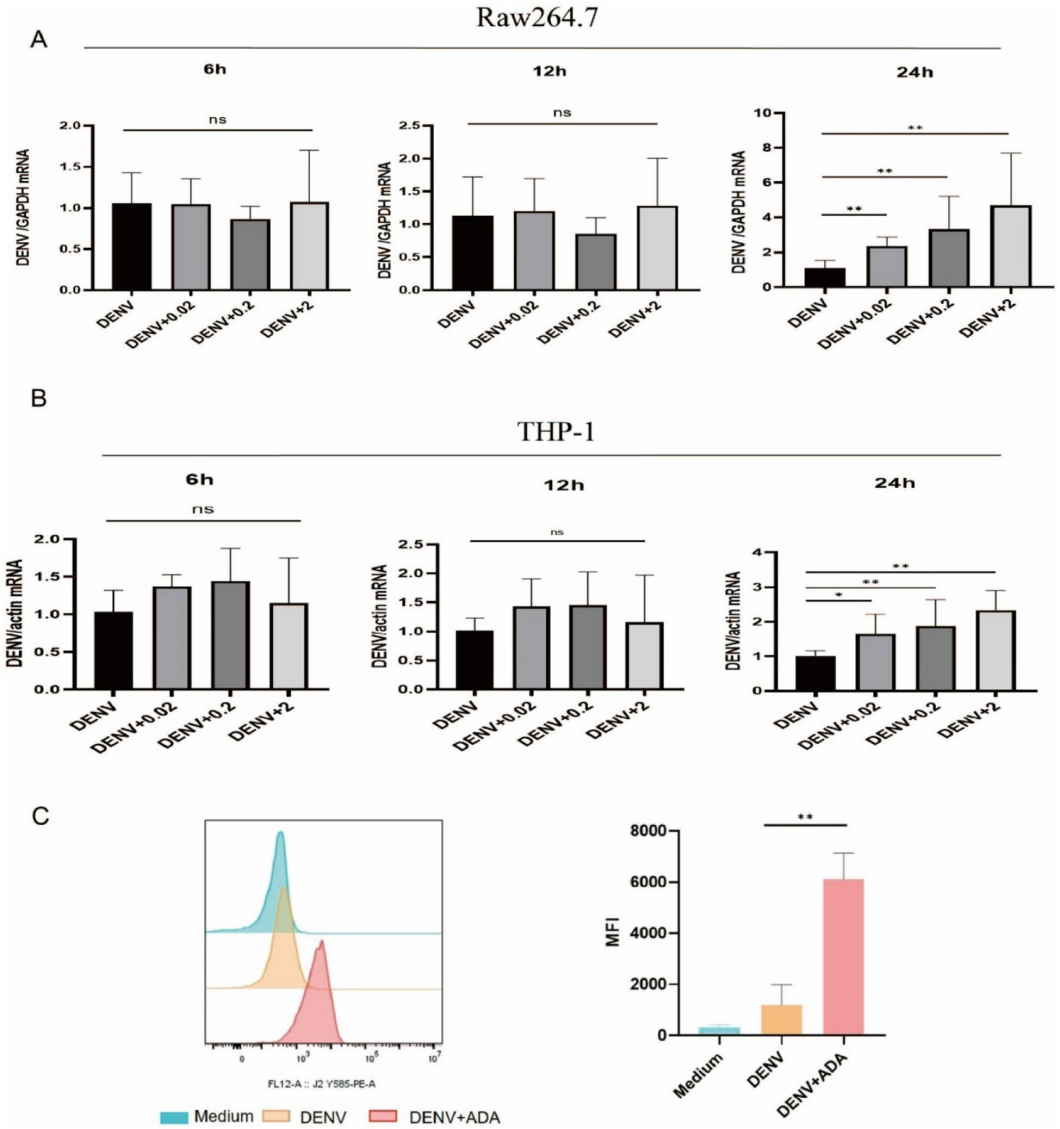
Effects of recombinant *Ae. albopictus* ADA on DENV-2 replication. Raw264.7 cells (**A**) and human THP-1 cells (**B**) were treated with DENV-2 (MOI = 0.25) alone or in combination with various doses (0.02, 0.2, and 2 μg/mL) of recombinant *Ae. albopictus* ADA. At various time points (6 h,12 h, and 24 h) following the stimulation, RNA was isolated from the cells, cDNA was generated, and qRT-PCR was performed to detect the genomes of the DENV envelope. Raw 264.7 cells treated with DENV-2 alone or in combination with recombinant *Ae. albopictus* ADA (2 μg/mL) and untreated group (Medium) were stained for dsRNA detection (J2 antibody); a flow cytometry (**C**) was applied to analyze the J2 fluorescence signal: representative result obtained from the flow cytometry analysis (left panel) and quantification of mean fluorescence intensity (MFI) (right panel) with n = 3. The data obtained from the analysis of Raw 264.7 cells were normalized to the mouse GAPDH using the ΔΔCT method and are presented as percentages of the average ΔΔCT value of the cells treated with DENV-2 alone; human THP-1 cells were normalized to human actin using the ΔΔCT method and are presented as percentages of the average ΔΔCT value of the cells treated with DENV-2 alone. A nonparametric Mann–Whitney test was applied for the statistical analysis. Labels: ns, insignificant (*p* > 0.05); *p < 0.05; **p < 0.01; ***p < 0.001. The data are representative of three independent experiments.

Mosquito saliva has been demonstrated to facilitate viral transmission. Therefore, to determine the potential role of ADA in DENV-2 infection, we incubated the DENV-2-infected Raw264.7 cells or THP-1 cells with or without rAb-ADA for 6 h, 12 h, and 24 h. Analysis of the expression of DENV-2 genes revealed that the viral load is higher in 24 h (Fig. S3) and the replication of the virus dose-dependently increased at 24 h in both cell lines (Fig. 6A,B). Further, Raw264.7 cells were stained for dsRNA (J2 antibody) detection, and flow cytometry was used to analyze the J2 fluorescence signal that signifies DENV replication. The results demonstrate the enhanced DENV replication in the presence of rAb-ADA (Fig. 6C). These findings suggest the potential involvement of ADA in the transmission of DENV-2 via facilitating DENV-2 replication.

### rAb-ADA regulates cytokine production during DENV-2 infection

**Figure 7:**
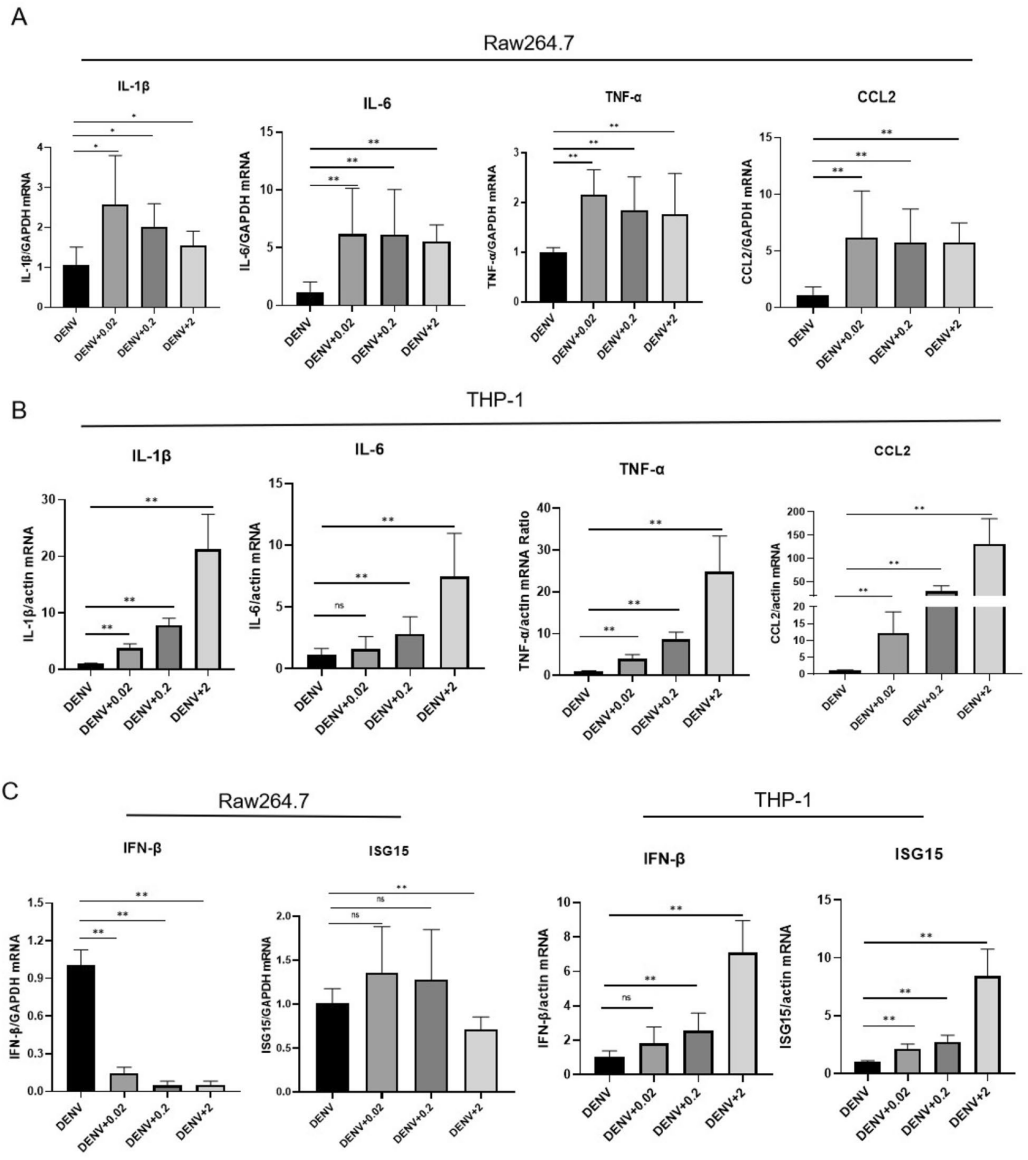
Recombinant *Ae. albopictus* ADA in combination with DENV-2 infection induces a substantial increase in the expression of cytokines. qRT-PCR analysis of cytokines (IL-1β, IL-6, TNF-α, CCL2, IFN-β, and ISG15) expression in Raw264.7 and human THP-1 after the infection with DENV-2 (MOI = 0.25) alone or in combination with various doses (0.02, 0.2 or 2 μg/mL) of recombinant *Ae. albopictus* ADA at 24 h. The data from Raw 264.7 cells were normalized to mouse GAPDH using the ΔΔCT method and are presented as percentages of the average ΔΔCT value of the cells treated with DENV-2 alone; human THP-1 cells were normalized to human actin using the ΔΔCT method and are presented as percentages of the average ΔΔCT value of the cells treated with DENV-2 alone. A nonparametric Mann–Whitney test was applied for the statistical analysis. Labels: ns indicates insignificant (*p* > 0.05); *p < 0.05; **p < 0.01; ***p < 0.001. The data are representative of three independent experiments.

Since DENV-2 induces cytokines production in macrophages and monocytes, it would be interesting to verify the immunomodulatory role of rAb-ADA during DENV-2 infection. Thus, we next determined the expression of cytokine in Raw264.7 and THP-1 cells infected with DENV-2 in the presence or absence of rAb-ADA for 24 h. Compared to infection without rAb-ADA, the co-administration of DENV-2 and rAb-ADA resulted in a substantial increase of inflammatory mediator genes IL-1β, IL-6, TNF-α, and CCL2 in Raw264.7 and THP-1 cells. However, the increment is dose-dependent in Raw264.7 but dose-dependent in THP-1 cells (Fig. 7A,B). The production of IL-1β, IL-6, and TNF-α in supernatant was also remarkably induced by rAb-ADA in DENV-infected cells (Fig. S3). Additionally, mRNA expression of IFN-β and ISG15 exhibited a different trend in Raw264.7 cells compared to the human THP-1 cells (Fig. 7C). The transcription level of IFN-β and ISG15 decreased in Raw264.7 cells incubated with rAb-ADA during DENV-2 infection. On the contrary, the transcription levels of IFN-β and ISG15 were enhanced in human THP-1 cells despite the same treatment conditions as Raw264.7 cells. These results suggest the modulation of cytokines released from DENV-2-infected macrophages and monocytes by ADA.

## Discussion

*Aedes albopictus* is currently ranked among the top 100 invasive species worldwide and can be found on all continents^37^. During probing and blood feeding, mosquitoes inject their saliva into the skin of vertebrate hosts^19^. Considering the roles of salivary proteins in inducing strong inflammatory immune responses at the bite sites, and promoting arboviruses transmission during blood feeding^38,39^, we compared the protein expression profiles in the saliva of blood-fed and unfed female mosquitos. Among the identified salivary proteins, ADA of *Ae. albopictus*, which can degranulate mast cells, was screened as a promising candidate. Then, the expression of ADA in the salivary glands of *Ae. albopictus* was verified via western blot. The analysis revealed a significant upregulation of ADA in female salivary glands following blood meal.

ADA is a crucial purine metabolic enzyme involved in the conversion of adenosine and 2′-deoxyadenosine into inosine and 2-deoxyinosine, respectively^40^. The enzyme activity of ADA has been demonstrated in the saliva of *C. quinquefasciatus* and *Ae. aegypti* (mosquitoes)^28^, *Glossina m. morsitans* (tsetse fly)^41^, *Lutzomiya longipalpis* (sand fly)^42^. This enzyme may also help blood feeding and regulate the expression of inflammatory cytokines^28,43,44^. Thus, we cloned and purified the recombinant *Ae. albopictus*-derived ADA and tested its enzyme activity. The results demonstrate the catalytic activity of ADA in the conversion of adenosine into inosine.

Studies have shown the interaction between viral components and mosquito saliva with the cells within the skin of the hosts, which are the first line of defense. Since these cells are permissive to infection and capable of eliciting immune responses, they play a dual role during the infection^45^. Therefore, a comprehensive understanding of the interaction between these cells, arbovirus, and mosquito saliva is important for developing strategies to combat mosquito-borne diseases. However, there are only a handful of studies investigating the interactions between certain salivary proteins and these cells^46^, particularly in *Ae. albopictus*.

A study reported the immunogenic role of ADA in *Ae. albopictus*^11^. Recently, we reported the interaction between ADA derived from the saliva of *Ae. albopictus* and mast cell-specific proteases including tryptase and chymase. The findings suggest the activation of mast cell-driven immune response by ADA, which leads to inflammation^15^. Nonetheless, other functions of ADA in *Ae. albopictus* remain unexplored. Macrophages, the monocyte-lineage cells, are antigen-presenting cells, which have an important role in immune response. To further investigate the potential function of ADA, we stimulated Raw264.7 macrophages and THP-1 monocytes with rAb-ADA. In this study, we identified ADA from *Ae. albopictus* as an immunomodulatory factor based on the increased levels of TNF-α, IL-6, IL-1β, TNF-α, IL-6, CCL2, IFN-β, and ISG15. Additionally, IL-1β, TNF-α, and IL-6 exerted pleiotropic effects on diverse cell types^47^. The major function of CCL2 production is to recruit cells^48^, which are important immune mediators involved in host inflammatory response. Our study further identified rAb-ADA as a positive regulator of NF-κB signaling, which targets TAK1. In general, the results demonstrate the role of rAb-ADA in modulating host inflammatory response via the activation of macrophages by targeting TAK1.

During viral transmission, both mosquito saliva and viruses are injected into the host. Some salivary proteins contribute to virus dissemination in the host by enhancing virus replication in host cells. Studies have found that ADA is highly expressed in the salivary glands of *Ae. aegypti* and contributes to DENV infection^30,31^. Another study reported the role of NeSt1 in promoting DENV and ZIKV replication in mice^49^. The current study revealed that the treatment of infected macrophages and monocyte cells with rAb-ADA leads to a high DENV-2 load. However, the role of salivary proteins in DENV replication and the associated mechanisms remain unclear. Recent studies have associated several key factors with DENV replication. In the infection models using mosquito cells, the double-stranded RNA (dsRNA) binding protein Loquacious, epigenetic regulator DIDO1, and septin 2 were found essential for the replication of DENV or ZIKV^50–52^. This finding sparks interest in determining the possible role of ADA in facilitating DENV-2 replication via modulating those factor-like proteins. The innate antiviral response to DENV is initiated by various immune cells and inflammatory mediators. Macrophages and monocytes are the main targets of DENV infection, whose functions include recognizing pathogen-associated molecular patterns (PAMPs) through pattern recognition receptors (PRRs)^53^. Toll-like receptors (TLRs) are the major PRRs that are coupled to detect specific viral components and induce the production of inflammatory cytokines, chemokines, and IFNs^54^. The effectors' action primarily results in antiviral responses, which could lead to disease pathogenesis should an imbalance in responses occur. In our studies, we observed that the mRNA expression level of IL-1β, IL-6, TNF-α, and CCL-2 was increased in both DENV-2-infected immune cells. Enhanced induction of IFN-β and ISG15 by rAb-ADA during DENV-2 infection was observed in THP-1 cells. Meanwhile, rAb-ADA suppressed the induction of IFN-β and ISG15 in Raw264.7 cells, suggesting the role of ADA in regulating cytokine production during DENV-2 infection. NF-κB is critical for TLR-mediated antiviral IFN responses and pro-inflammatory activation. These findings demonstrate that rAb-ADA is a positive regulator of NF-κB signaling. However, the mechanisms of ADA-altered cytokine expression in facilitating DENV-2 replication via targeting the NF-κB signaling require further investigation.

## Conclusions

In this work, distinction protein spots in the salivary gland of female *Ae. albopictus* were modulated by blood-feeding. The results demonstrate the upregulation of ADA as evidenced in WB. Moreover, enhanced expression of ADA and its enzymatic activity in the salivary gland of female *Ae. albopictus* was detected. Subsequently, a rAb-ADA protein with ADA enzymatic activity was obtained. Further investigation revealed the induction of proinflammatory cytokines by rAb-ADA via targeting TAK1, facilitating DENV-2 replication in Raw264.7 and THP-1 cells. The results demonstrate the possible role of ADA as an immunomodulatory factor that enhances DENV-2 replication in immune cells. These findings will help establish the role of *Ae. albopictus* salivary protein in arboviruses transmission, and may provide a theoretical basis for preventing DENV transmission in nature.

## Methods

### Mosquitoes, cells, virus

*Aedes albopictus* mosquitoes (Guangzhou strain) were kindly provided by Prof. Zhao Tongyan (Beijing Institute of Microbiology and Epidemiology) and reared as previously described^55^. Raw264.7 cells were purchased from the Center for Type Culture Collection (Wuhan, China) and cultured in Dulbecco’s modified Eagle’s medium (DMEM; Gibco) supplemented with 10% heat-inactivated fetal bovine serum (FBS; Gibco), streptomycin (100 mg/mL), and penicillin (100 U/mL) and maintained at 37 °C (5% CO_2_). THP-1 cells were purchased from the National Collection of Authenticated Cell Cultures (Shanghai, China) and maintained in Roswell Park Memorial Institute (RPMI) 1640 medium (Gibco) supplemented with 10% heat-inactivated fetal bovine serum (FBS; Gibco), streptomycin (100 mg/mL), and penicillin (100U/mL) and maintained at 37 °C (5% CO_2_). DENV-2 strain was propagated in *Ae. albopictus* C6/36 cells. The virus titers were determined by a plaque formation assay.

### Preparations of *Ae. albopictus* salivary gland protein extracts

Salivary glands were dissected from adult female mosquitoes on day five post-emergence (PE) of unfed and blood-fed groups. These salivary glands were re-suspended in 100 μL PBS and kept at − 80 °C until further use. Salivary glands were disrupted using a tissue homogenizer (MIULAB, China) for 10 min on ice and were centrifuged at 12,000*g* for 45 min. The supernatant was kept at − 80 °C until further use.

### Two-dimensional gel electrophoresis and MALDI-TOF MS analysis

Salivary proteins were desalted using a 2-D Clean-Up kit (GE Healthcare, USA) and quantified using a Micro BCA Protein Assay Kit (Pierce, Rockford, IL). Proteins of 300 μg were loaded on an Immobiline DryStrip (pI 3–10, 24 cm, Bio-Rad, USA) to perform the first dimension isoelectric focusing separation. Following 14 h of rehydration, the strips were focused using Ettan IPGphor III (GE Healthcare, USA) according to the manufacturer's instructions. The focused strips were then applied to the SDS-PAGE for the second dimension. After the electrophoresis, the gels were stained using Coomassie Brilliant Blue (CBB) and viewed using ImageScanner III (GE Healthcare, USA). The images were analyzed using ImageMaster 2D Platinum 6.0 software (GE Healthcare, Buckinghamshire, UK). The molecular weight (MW), by which the isoelectric point (pI) and normalized volume of each protein spot from unfed or blood-fed gel samples were calculated following the software manuals supplied by the manufacturer. The average normalized volume (ANV) of each major protein in the unfed and blood-fed groups was obtained. The fold expression of ≥ 2 of the major proteins was calculated and compared between the two 2-DE gel images.

Differentially expressed protein spots were selected and excised from the 2-D gels and subjected to in-gel digestion. A sample of 1 μL in volume was spotted onto a MALDI-TOF MS plate with 0.5 μL of a-cyano-4-hydroxycinnamic acid (a-CHCA) for analysis by 4800 Plus MALDI TOF/TOFTM analyzer (Applied Biosystems, USA).

### Enzyme assays

Measurement of adenosine deaminase activity was performed in quartz microcuvettes via a spectrophotometric approach. Adenosine of 1 mM in a buffer containing 10 mM Hepes and 150 mM NaCl (HS, pH = 7.0) was added to the cuvette, followed by the addition of 10 μL of salivary gland homogenates extracted from 20 pairs of female salivary glands on day five PE or 0.67 μM purified rAb-ADA expressed in the *Escherichia coli* BL21 (DE3) strain. After mixing the sample in the solution, the absorbance at 220 and 300 nm was continuously measured 30 times by using a UV-2600 spectrophotometer (Shimadzu, Japan).

### Prokaryotic expression and purification of recombinant *Ae. albopictus* ADA

ADA from *Ae. albopictus* was expressed and purified as described previously^15^. A DNA fragment encoding a mature ADA (GenBank: AAV90660.1) was amplified by PCR and inserted into the cloning site of the pET32a(+) vector. *E. coli* BL21 (DE3) cells were transformed with the recombinant plasmid (pET32a(+)-ADA) and 1 mM isopropyl-β-d-thiogalactosidase (IPTG) was added to the Luria–Bertani (LB) medium to induce rAb-ADA expression. rAb-ADA protein was purified using His Bind Purification Kit (Novagen) according to the manufacturer’s instructions and dialyzed against 1× PBS buffer at 4 °C overnight. The purified protein was analyzed on 10% sodium dodecyl sulfate–polyacrylamide gel electrophoresis (SDS-PAGE) and visualized using Coomassie brilliant blue R-250. The absence of endotoxin was tested using an ELISA Kit for Lipopolysaccharides as reported in Ref^15^.

### Production of rabbit anti-ADA polyclonal antibody

rAb-ADA of 400 μg was mixed with Freund's Complete Adjuvant (Sigma, USA) at a ratio of 1:1. The suspension was subcutaneously injected at various sites on the back of a 15-week-old male New Zealand rabbit during the first immunization. The subsequent subcutaneous injections with a half-dose of rAb-ADA mixed with incomplete Freund’s adjuvant (Sigma, USA) at a ratio of 1:1 was performed every week. Rabbit blood was collected after the fourth immunization and stored at − 20 °C until further use.

### Western blotting

Samples were separated on 10% SDS-PAGE and subsequently transferred to the Immobilon-NC Transfer Membrane (Millipore, USA). The membranes were blocked with 5% milk in Tris-buffered saline overnight and subsequently incubated with the following primary antibodies for 2 h at room temperature: anti-p65(8243S, CST); anti-phospho-NF-κB p65 (3033S, CST), anti-IκBα (4812S, CST), anti-p-IκBα (2859S, CST), anti-β-actin (3700S, CST), anti-His (T0009, Affinity), anti-ADA polyclonal**.** After the incubation, membranes were washed three times and incubated with a secondary HRP conjugated goat anti-rabbit (SA0001-2, proteintech) or goat anti-mouse (RS0001, Immunoway) (at 1:2000 dilution) for 1 h at 37 °C. The signals corresponding to the proteins were detected by enhanced chemiluminescence (ECL) (Millipore, USA). All the blots were cut prior to hybridisation with antibodies.

### rAb-ADA-stimulation in THP-1 and Raw264.7 cells

The THP-1 and Raw264.7 cells were seeded at 25,000 cells/well in 24-well plates and maintained at 37 °C (5% CO_2_) for approximately 12 h until 70% confluence was reached. The THP-1 cells were treated with various doses (0.02, 0.2, and 2 μg/mL) of rAb-ADA. The cells were harvested at different time points (6 h, 12 h, and 24 h) post-stimulation to determine the mRNA expression of IL-1β, IL-6, TNF-α, CCL2, IFN-β, and ISG15 via qRT-PCR. The Raw264.7 cells were treated with various doses (0.02, 0.2, and 2 μg/mL) of recombinant *Ae. albopictus* ADA was harvested at 24 h, and the supernatants were collected and kept at − 80 °C until further use.

### The infection of Raw264.7 and THP-1 cells with DENV-2

The Raw264.7 and THP-1 cells were seeded at 25,000 cells/well in 24-well plates and maintained at 37 °C (5% CO_2_) for approximately 12 h until 70% confluence was reached. The cells were either treated with DENV-2 0.25 multiplicity of infection (MOI) alone or in combination with various doses (0.02, 0.2, and 2 μg/mL) of rAb-ADA for 6 h,12 h and 24 h. After the incubation, the cells were harvested to determine viral replication and mRNA expression of IL-1β, IL-6, TNF-α, CCL2, IFN-β, and ISG15 via qRT-PCR.

### Quantitative real-time PCR

The total RNA was isolated from tissues and cells by using TRIzol reagent (Invitrogen, USA) and reverse-transcribed into cDNA using PrimeScript™ RT reagent Kit with gDNA Eraser (TaKaRa, Japan) according to the manufacturer’s protocol. Real-time qRT-PCR was performed on Bio-Rad CFX-96 Connect Real-Time Detection System to measure the mRNA levels of tested genes. Primer sequences are shown in Supplementary Table 1. The 2^−∆∆^CT method was used to determine relative expression levels, and A nonparametric Mann–Whitney test was used to determine if the differences were statistically significant. All analyses were performed using the GraphPad Prism statistical software (Version8.0.2).

### Enzyme-linked immunosorbent assay (ELISA)

IL-1β, IL-6, and TNF-α in supernatants of Raw264.7 mouse macrophage were detected by using mouse IL-1β, IL-6, and TNF-α ELISA kit (Invitrogen) according to the manufacturer’s instructions.

### Intracellular staining for flow cytometry

Raw264.7 cells were first fixed for 30 min and further permeabilized for 30 min using eBioscience™ Intracellular fixation & permeabilization buffer set (88-8824-00, Thermo Fisher Scientific, USA). The cells were washed twice with PBS + 1% FBS. Next, the Raw264.7 cells were blocked for 30 min with PBS + 5% FBS, washed twice, and stained with dsRNA using J2 antibody (10010200, Nordic-MUbio) (1:500 dilution) for 1 h, followed by three cycles of washing with PBS + 1% FBS. The cells were then stained with a secondary antibody, Goat anti-Mouse IgG conjugated with PE (12–4010-82, Invitrogen), at 1:1500 dilution for 1 h in PBS + 5% FBS, followed by three cycles of washes. The cells were finally resuspended in PBS + 1% FBS for analysis on Cytoflex LX (Beckman, USA).

### Ethical approval

New Zealand rabbit were provided by the Animal Center of Guizhou Medical University and was cultured in a pathogen-free environment. Animal experiments was conducted and approved by the Animal Care welfare Committee of Guizhou Medical University under protocol number 1603140 and all methods were carried out in accordance with relevant guidelines and regulations. This study is reported in accordance with ARRIVE guidelines (https://arriveguidelines.org).

### Supplementary Information

Supplementary Information.

## Data Availability

Data supporting the conclusions of this article are included within the article and its additional files. The raw datasets used and analysed during the present study are available from the corresponding author upon reasonable request.
