# Supplementary material for: Aedes albopictus salivary adenosine deaminase is an immunomodulatory factor facilitating dengue virus replication
Source: Sci Rep. 2023 Oct 4;13:16660. doi: 10.1038/s41598-023-43751-1 (PMC10551004; doi:10.1038/s41598-023-43751-1)
Supplement: Supplementary file 1 — Supplementary Information. [file 41598_2023_43751_MOESM1_ESM.pdf]

Aedes albopictus salivary adenosine deaminase is an immunomodulatory factor facilitating dengue virus replication

Xiaohui Mu[1,2], Zimin Lin[1], Yu Sun[3], Lu Chen[3], Qingqiao Lv[4], Cejuan Ji[5], Xiaoyuan Kuang[1], Weiyi Li[1], Zhengling Shang[6], Jinzhi Cheng[1], Ying Nie[1], Zhiqiang Li [6,] *and Jiahong Wu[1,] *

Supplementary Table 1: List of Primers

Primers for qRT-PCR	*Upper primer*	*Lower primer*
Mouse-GAPDH	*TGGCCTTCCGTGTTCCTAC*	*GAGTTGCTGTTGAAGTCGCA*
Mouse-TNF-α	*GACCCTCACACTCAGATCAT*	*TTGAAGAGAACCTGGGAGTA*
Mouse-IL-6	*GAGGGATACCACTCCCAACAGAC*	*AAGTGCATCATCGTTGTTCATACA*
Mouse-IL-1β	*CAACCAACAAGTGATATTCTCCATG*	*GATCCACACTCTCCAGCTGCA*
Mouse-IFN-B	*GCCTTTGCCATCCAAGAGATGC*	*ACACTGTCTGCTGGTGGAGTTC*
Mouse-ISG15	*TGACTGTGAGAGCAAGCAGC*	*CCCCAGCATCTTCACCTTTA*
Mouse-CCL-2	*CACTCACCTGCTGCTACTCATTC*	*GGTGCTGAAGACCTTAGGGC*
DENV Envelope gene	*CAGATCTCTGATGAATAACCAACG*	*CATTCCAAGTGAGAATCTCTTTGTCA*
Human Actin	*TGACGTGGACATCCGCAAAG*	*CTGGAAGGTGGACAGCGAGG*
Human IL-1β	*TCTTCTCGAACCCCGAGTGA*	*CCTCTGATGGCACCACCAG*
Human IL-6	*AAACAACCTGAACCTTCCAAAGA*	*GCAAGTCTCCTCATTGAATCCA*
Human TNF-α	*TCTTCTCGAACCCCGAGTGA*	*CCTCTGATGGCACCACCAG*
Human CCL2	*CGCCTCCAGCATGAAAGTCT*	*GGAATGAAGGTGGCTGCTATG*
Human IFN-β	*CCAACAAGTGTCTCCTCCAAATT*	*GTAGGAATCCAAGCAAGTTGTAGCT*
Human ISG15	*AGGACAGGGTCCCCCTTGCC*	*CCTCCAGCCCGCTCACTTGC*
Aedes albopictus ADA	*CGGAGGTTTACGACTTGGATG*	*GTAGATGAACTTGACGCCGATG*
Aedes albopictus RPS6	*GAAGTTGAACGTATCGTTTC*	*GAGATGGTCAGCGGTGATTT*

Supplementary Fig.S1

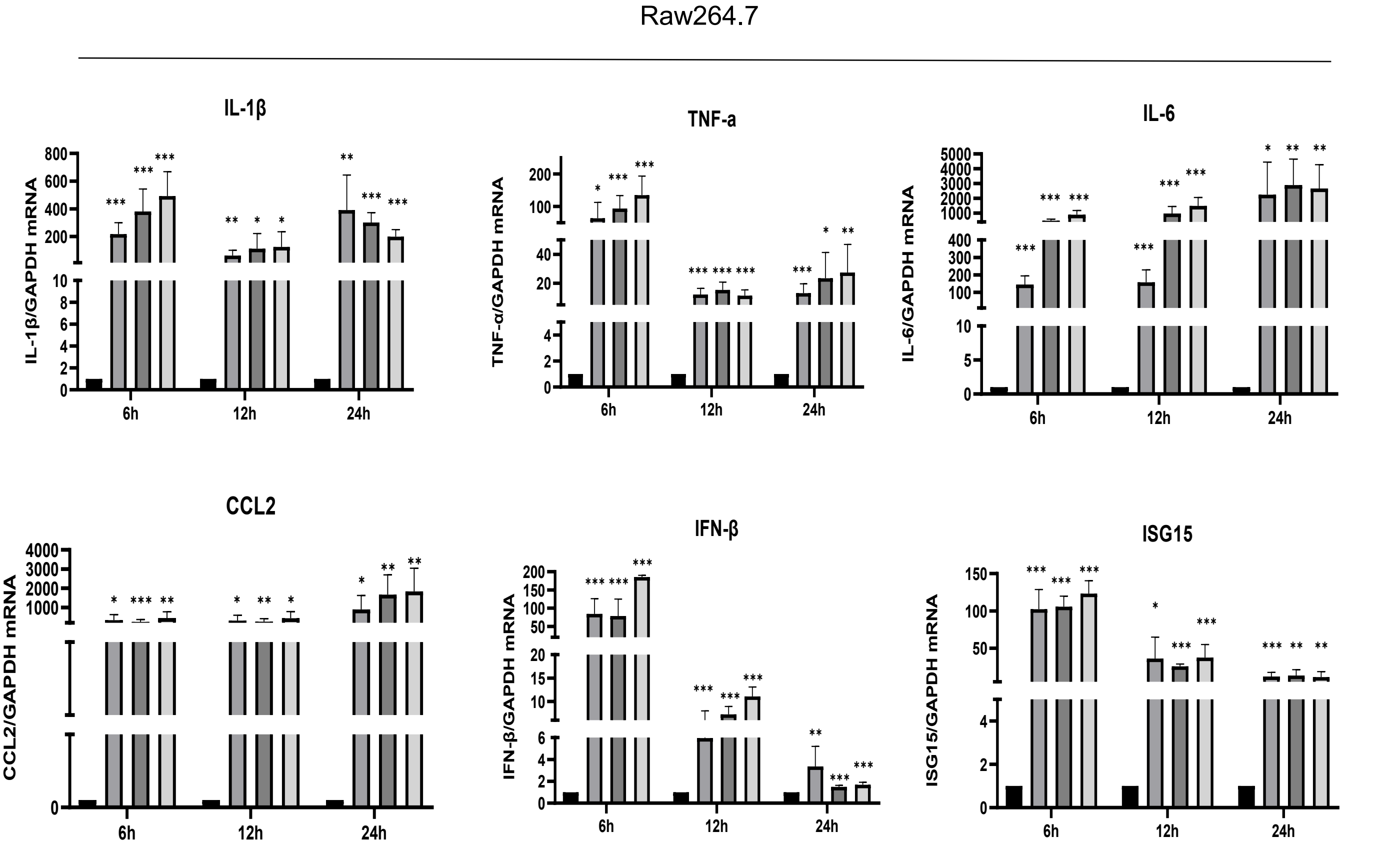

Fig. S1| Recombinant Ae. albopictus* ADA promote cytokines production in Raw264.7 Cells.** *Raw264.7 Cells were treated with various doses (0.02, 0.2 or 2 μg /mL) of recombinant Ae. albopictus ADA. After various times(6h,12h or 24h) stimulation, RNA was isolated from cells, cDNA was generated, and qRT-PCR was used to measure the mRNA lever of IL-1β,IL-6,TNF-α,CCL2,IFN-β,ISG15. The data were normalized to mouse GAPDH with the ΔΔCT method and are presented as percentages of the average ΔΔCT value of no-treated(PBS) cells. A nonparametric Mann–Whitney test was used for the statistical analysis.ns,no significant($p > 0.05$); *$p < 0.05$; **$p < 0.01$;$p < 0.001$.The data were combined based on three independent experiments.*

Supplementary Fig.S2

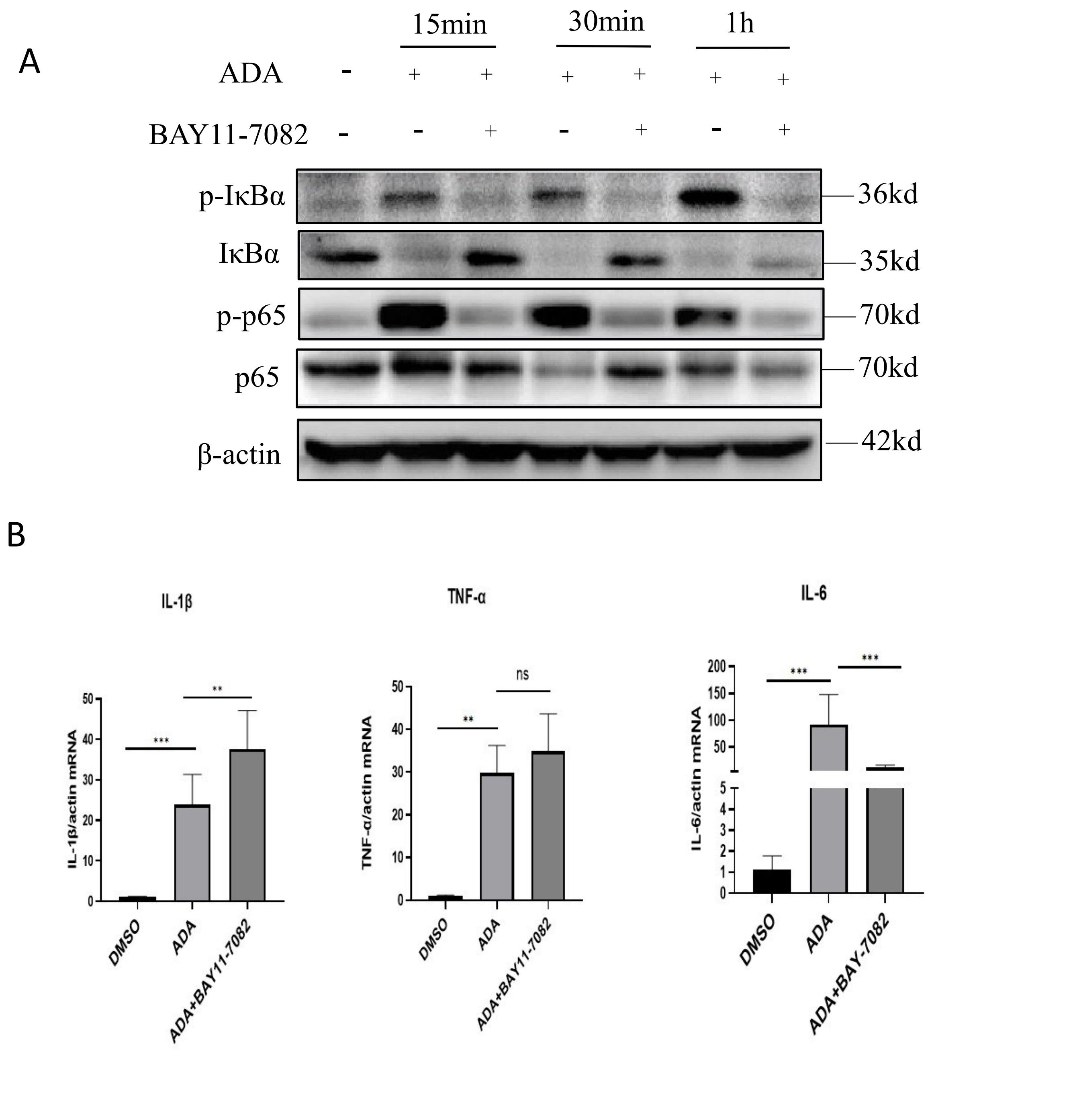

Fig.S2| The influence of inhibitor BAY11-7082 on NF-κB pathway which activated by rAb-ADA*.(A) Human THP-1 cells treated with inhibitor BAY11-7082(10 μM) or DMSO for 3h before stimulated with ADA (2 μg /mL) for various times(15min,30min or 1h), p-IκBα, IκBα, p-p65 and p65 were analysied by Immunoblot. (B) Human THP-1 cells treated with inhibitor BAY11-7082 (10 μM) or DMSO for 3h before stimulated with ADA (2 μg /mL) for 24h,then RNA was isolated from cells, cDNA was generated, and qPCR was used to measure the mRNA lever of IL-1β,IL-6,TNF-α.A nonparametric Mann–Whitney test was used for the statistical analysis ($p < 0.05$).*

Supplementary Fig.S3

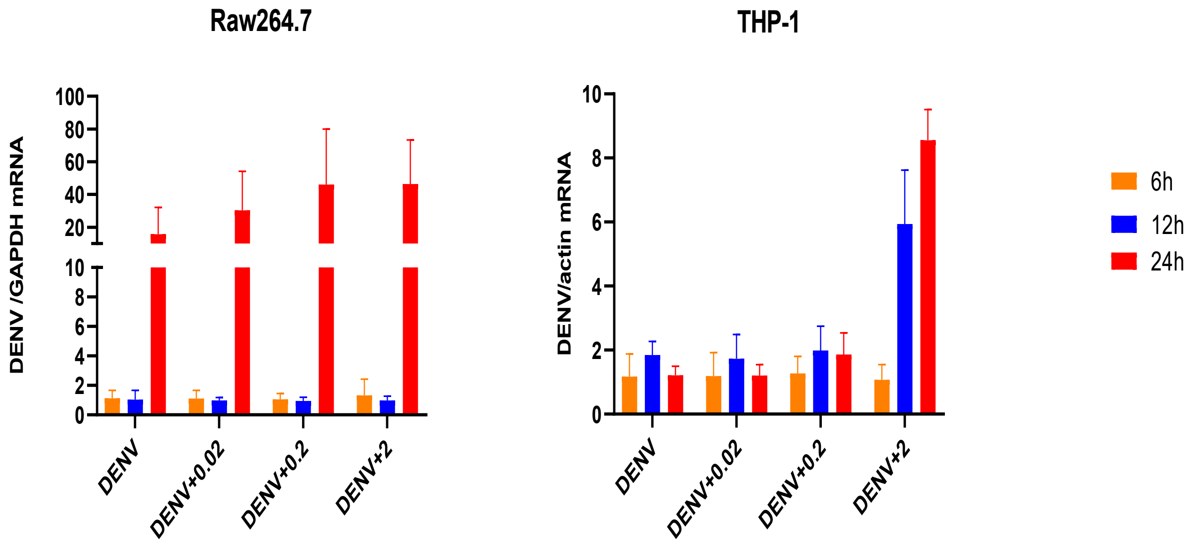

Fig.S3| the viral load in the cell at different time point. *Raw264.7 Cells and THP-1 cells were treated with various doses (0.02, 0.2 or 2 μg /mL) of recombinant Ae. albopictus ADA. After various times(6h,12h or 24h) stimulation, RNA was isolated from cells, cDNA was generated, and qRT-PCR was used to measure the mRNA lever of DENV-2envelope at different time point. The data were normalized to mouse GAPDH or human actin with the ΔΔCT method.*

Supplementary Fig.S4

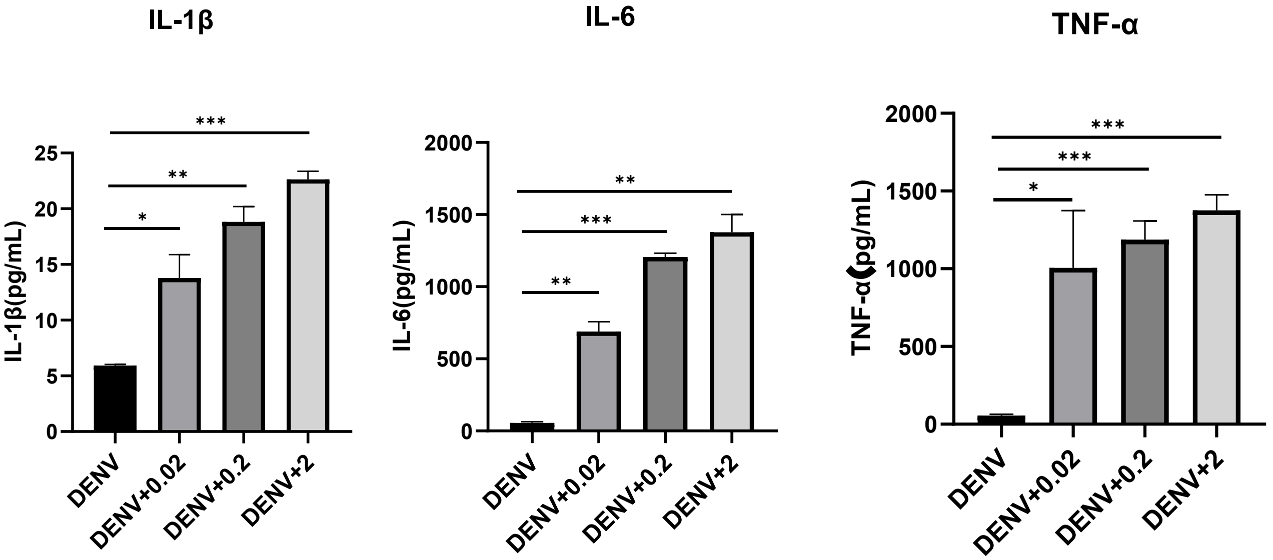

Fig.S4| Recombinant Ae. albopictus **ADA** ***and DENV-2 infection combine to induce a substantial increase production of IL-1β,IL-6 and TNF-α.*** *ELISA of IL-1β,IL-6 and TNF-α in supernatants of Raw 264.7 cells treated with various doses (0.02, 0.2 or 2 μg /mL) of recombinant Ae. albopictus* ADA *at 24h. A nonparametric Mann–Whitney test was used for the statistical analysis.ns,no significant($p > 0.05$); *$p < 0.05$; **$p < 0.01$;***$p < 0.001$.The data were combined based on three independent experiments.*

Supplementary: Figue 2B full-length gels

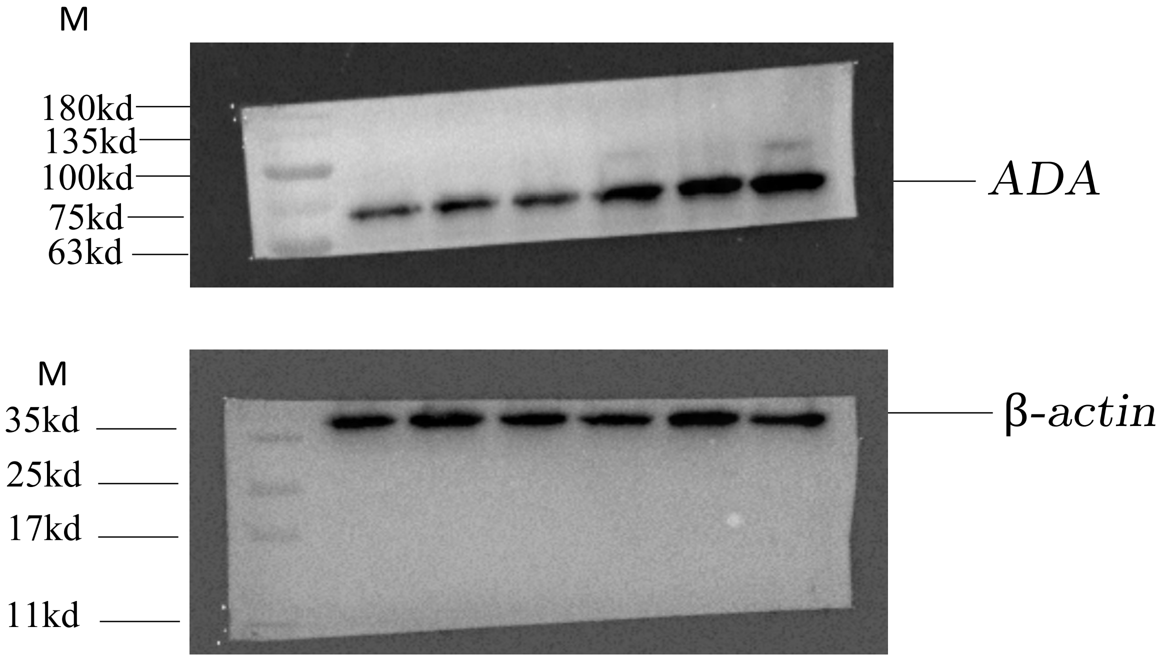

Figue 2B full-length gels*: Salivary glands were collected at three different times from 5-days old female mosquitoes with (BF: left three lane) or without (UF:right three lane) blood-fed and the expression of salivary ADA was detected by immunoblotting with anti-ADA antibody. M:marker (Solarbio, China)*

Supplementary: Figue 5A full-length gels

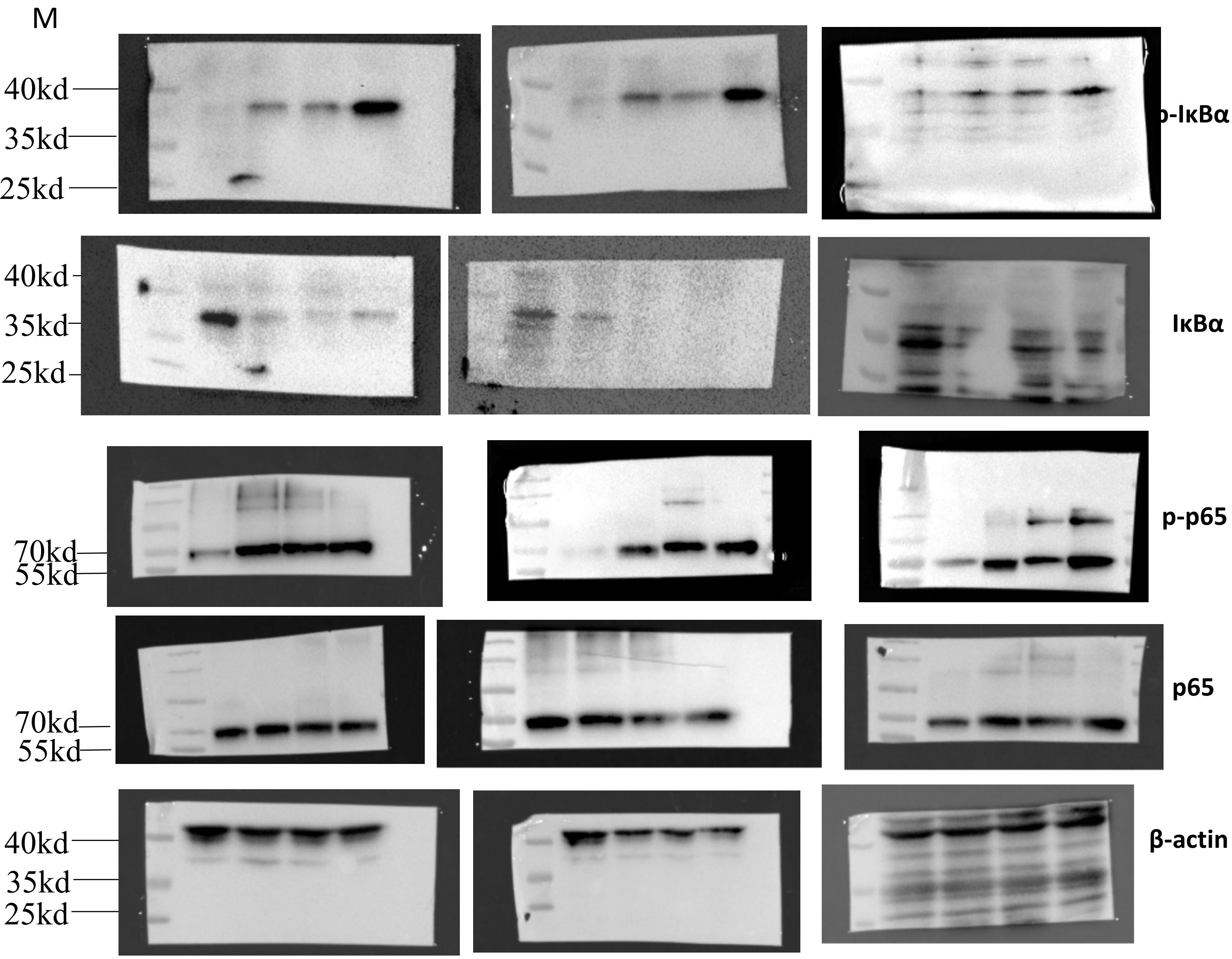

Figue 5A full-length gels: *Immunoblot analysis of phosphorylated IκBα(p-IκBα), total proteins of IκBα(IκBα), phosphorylated p65(p-p65) and p65 in human THP-1 cells which untreated (Med) or treated with ADA (2 μg /mL) at various times (15min,30min or 1h). Each image represents a repeated experiment. M:marker (Thermo, USA)*

Supplementary: Figue 5C full-length gels

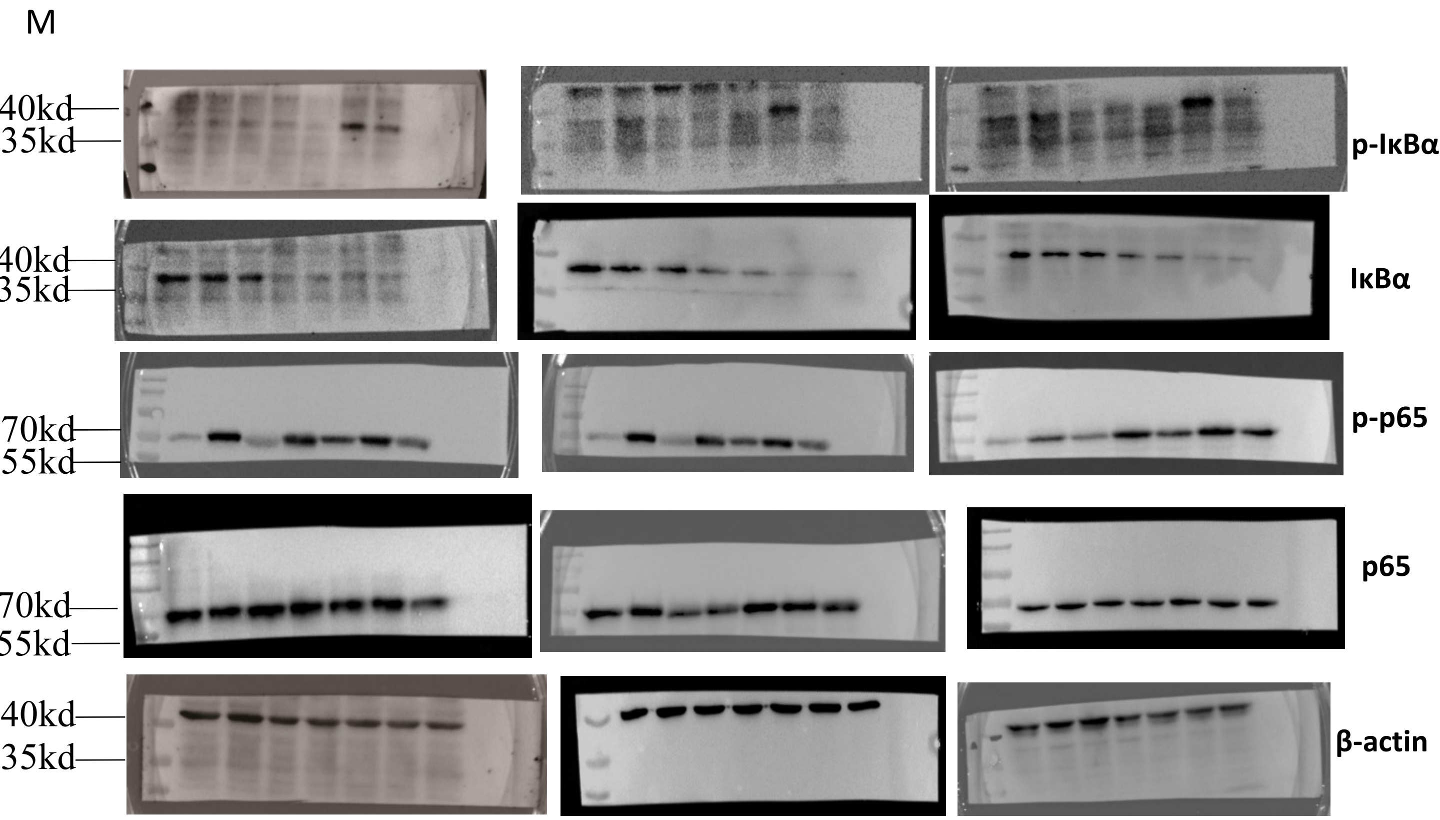

Figue 5C full-length gels: *Human THP-1 cells treated with inhibitor Takinib(10μM) or DMSO for 3h before stimulated with ADA (2 μg/mL) for various times (15min,30min or 1h), p-IκBα, IκBα, p-p65and p65 were analysied by Immunoblot.Each image represents a repeated experiment. M:marker (Thermo, USA)*

Supplementary: Figue S2A full-length gels

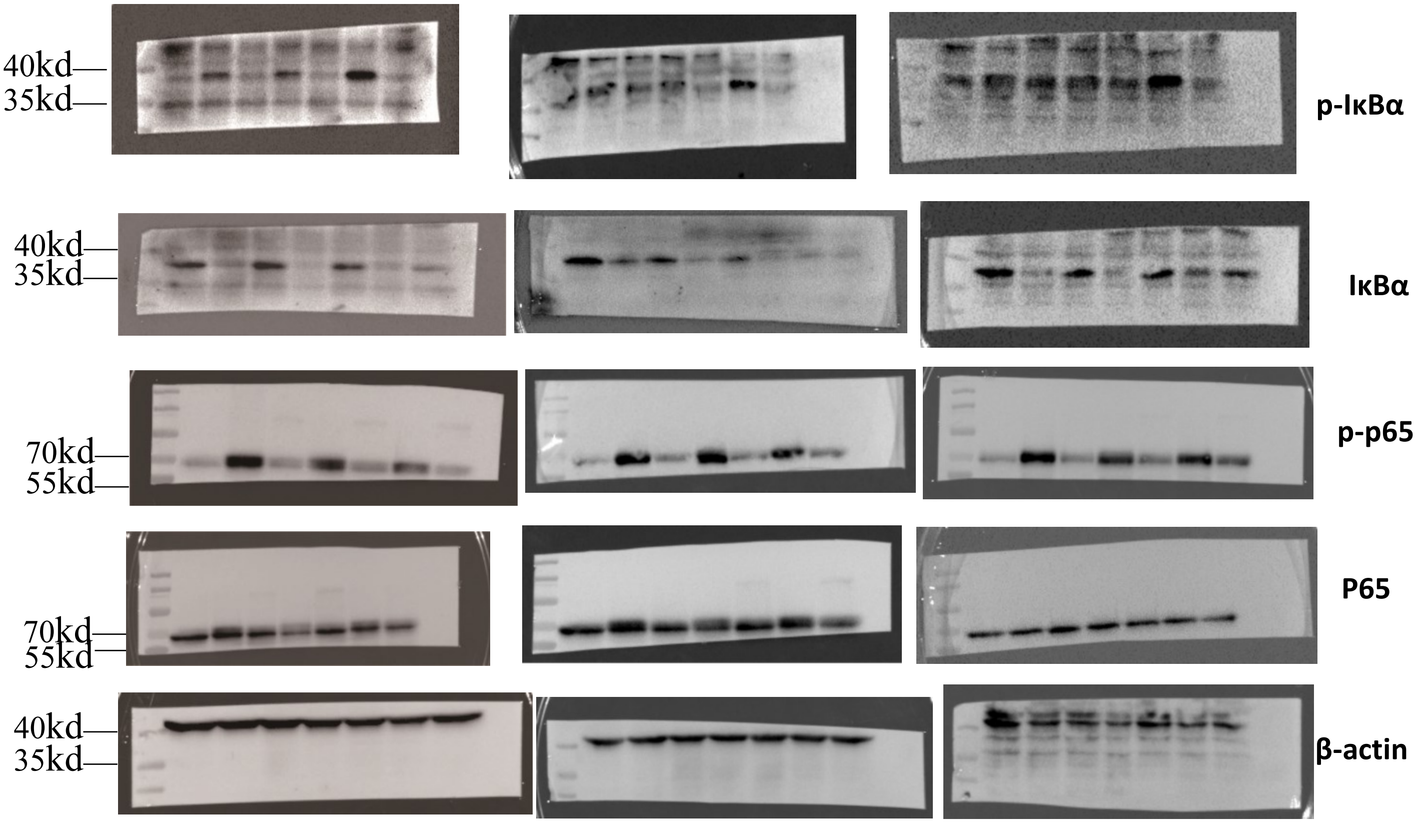

Figue S2A full-length gels: *Human THP-1 cells treated with inhibitor BAY11-7082(10 μM) or DMSO for 3h before stimulated with ADA (2 μg /mL) for various times(15min,30min or 1h), p-IκBα, IκBα, p-p65 and p65 were analysied by Immunoblot. Each image represents a repeated experiment. M:marker (Thermo, USA)*
